# Supplementary material for: Active site specificity profiling datasets of matrix metalloproteinases (MMPs) 1, 2, 3, 7, 8, 9, 12, 13 and 14
Source: Data Brief. 2016 Feb 22;7:299–310. doi: 10.1016/j.dib.2016.02.036 (PMC4777984; doi:10.1016/j.dib.2016.02.036)
Supplement: Supplementary file 10 — Supplementary material [file mmc10.zip › WebPICS_hMMP3_G_1%/P3.html]

 

PICS results


|  |  |
| --- | --- |
| **P3\_A**  20 in 151 sites   13.2 %    effects > 10 perc. pnts.  (vice-versa in brackets)  P1prime\_C: 13.4 (26.8)   P1prime\_V: 11.1 (10.6)   P2\_L: 13.7 (16.2)   P2prime\_L: 27.4 (28.9) |  |
| **P3\_I**  13 in 151 sites   8.6 %    effects > 10 perc. pnts.  (vice-versa in brackets)  P1\_N: 45.6 (24.7)   P1prime\_V: 24.6 (15.2)   P2\_H: 11.4 (24.7)   P2\_L: 34.9 (26.7)   P2prime\_H: 12.1 (31.4)   P2prime\_R: 14.5 (14.5)   P2prime\_T: 16.5 (21.4)   P3prime\_A: 21.3 (10.6) |  |
| **P3\_L**  17 in 151 sites   11.3 %    effects > 10 perc. pnts.  (vice-versa in brackets)  P2\_C: 13.0 (31.6)   P2\_S: 14.9 (19.5)   P2prime\_H: 14.3 (48.7)   P2prime\_Q: 11.0 (18.7)   P3prime\_A: 29.9 (19.5) |  |
| **P3\_P**  43 in 151 sites   28.5 %    effects > 10 perc. pnts.  (vice-versa in brackets)  P1\_N: -13.6 (-24.3)   P2\_Q: 11.6 (35.8)   P3prime\_A: -10.2 (-17.0) |  |
| **P3\_V**  25 in 151 sites   16.6 %    effects > 10 perc. pnts.  (vice-versa in brackets)  P2\_A: 20.8 (17.9) |  |
